# Analysis of the interprovincial embodied carbon flow network of China’s exports

**DOI:** 10.1371/journal.pone.0275286

**Published:** 2022-10-11

**Authors:** Zhipeng Tang, Haojie Yu, Jialing Zou

**Affiliations:** 1 Institute of Geographic Sciences and Natural Resources Research, Chinese Academy of Sciences, Beijing, China; 2 College of Resources and Environment, University of Chinese Academy of Sciences, Beijing, China; 3 Guangdong Institute for International Strategies, Guangdong University of Foreign Studies, Guangzhou, China; University College Cork National University of Ireland, IRELAND

## Abstract

We calculate the embodied carbon emissions of China’s through the multiregional input–output (MRIO) method, then we construct the interprovincial embodied carbon flow networks of China’s exports based on the mean threshold, and the application of complex network analysis to conduct a detailed examination of the overall characteristics, key nodes and edges, and community structure of China’s interprovincial embodied carbon flow network. We extended the embodied carbon flow network analysis at the provincial level. The results demonstrated the following: (1) The interprovincial embodied carbon flow network of China’s exports has small-world and scale-free characteristics. The node degree probability distribution curves for the networks obviously conformed to a decreasing power law distribution, indicating that a few industrial sectors carry a large amount of embodied carbon and suggesting that reducing the embodied carbon of China’s exports could yield twice the results with half the effort as long as attention is paid to a few sectors. (2) The key nodes and edges in the networks show that industrial sectors and production chains such as the power and heat production and supply industry, the petroleum processing, coking, and nuclear fuel processing industry, and the metal smelting and calendering industry play the role of key “bridges” in the entire network, among which Guangdong, Hebei, Jiangsu, Inner Mongolia, and Shanxi are important node provinces and the main flow paths for the generation of embodied carbon in national exports. These industrial sectors and production chains should bolster their policies to encourage the innovation of carbon emission reduction technologies and decrease carbon emissions, so as to reduce the embodied carbon of national exports on a large scale. (3) The number of communities firstly increased then decreased from 2007 to 2017, while the aggregation coefficient of the node and correlation density within first community displayed firstly downward then upward trends, reflecting firstly decentralization then centralization of the interprovincial embodied carbon flow.

## 1. Introduction

Climate change has become a global hot issue that has aroused the concern of governments around the world. Since China’s economic reform and opening up, its foreign trade has developed rapidly. According to the constant price calculations of the United Nations Statistics Division in 2015, the average annual export growth rate from 1980 to 2018 was 13.4%, which was 3.9% higher than the GDP growth rate over the same period, thus making exports one of the main engines of economic growth. However, China’s export of processing trade accounts for a relatively high proportion, and the energy resources invested by provinces, regions, and cities are also high. Moreover, China’s emergence as a “world factory” has been accompanied by severe environmental pollution. According to scholars from the National Center for Atmospheric Research in the United States, approximately 7–14% of China’s carbon emissions from 1997 to 2003 were produced for American consumers [1]. In 2007, China’s Ministry of Commerce and General Administration of Customs jointly announced new restrictions on the processing trade aimed at strictly controlling the export of “heavy energy consumption, heavy pollution and resource-related product” products, which were revised and added to in 2009 and 2010. The long-term dynamic relationships between the export trade and energy consumption, carbon emissions, and other pollutants have attracted the attention of numerous scholars around the world [2–11]. The concept of embodied carbon provides a scientific basis for the comprehensive accounting of carbon emissions in trade commodities [12]. During the production of national export commodities, the embodied carbon of intermediate products produced by interregional industrial linkages flows along multiple production links to form a complex network. In 2016, China officially signed the Paris Agreement, which promised to reduce carbon emissions per unit of GDP by 60–65% by 2030 compared with 2005. In 2020, the Chinese government once again stressed the ambition of carbon emission reduction and promised to strive to achieve the arduous task of carbon peak by 2030 and carbon neutralization by 2060. These national long-term development goals need to be distributed to all provinces. The fact that the embodied carbon flow between provinces makes the emission reduction goal of any province will affect the implementation of other provinces, which makes the task of reducing the implicit carbon in exports become very complex. Overall, behind the national export chain, the total amount of embodied carbon flow in the production networks formed by domestic provinces plays a decisive role in China’s low-carbon export goals. In addition, different forms of interprovincial embodied carbon flow networks play an important role in the high-quality development of China’s exports. Therefore, it is crucial to analyze the structural characteristics of the interprovincial embodied carbon flow networks and identify the role of groups in these networks, which will assist the rational and scientific design of interregional collaborative emission reduction policies and lay a foundation for further clarifying the responsibilities of each region in reducing carbon emissions.

In terms of research into embodied carbon flow, the current literature mainly reveals the phenomenon of carbon transfer between regions in combination with the multiregional input–output method [13–17]. It should be noted that the multiregional input–output analysis method is suitable for realizing a detailed analysis of the embodied carbon flow directions of specific regional industrial sectors, but it fails to reveal the interaction relationships between sectors from the whole industrial system [18]. In complex network theory, a complex network is a set of nodes with independent characteristics that are interconnected with other individuals. It has small-world and scale-free characteristics. Each individual can be regarded as a node in the graph, and the interconnections between nodes can be regarded as edges [19]. Complex network theory can be applied to determine the network characteristics of complex systems as a whole and provide internal information regarding relevant components of the system. This theory has been extensively used in the resource and environment fields [20–22]. In terms of embodied carbon flow, Duan and Jiang studied the characteristics of trade embodied carbon flow around the world from 1995 to 2011 based on the World Input–Output Database [22], while Jiang et al. described the major characteristics of the global embodied carbon emission transfer network from 2000 to 2015 by using the Eora database and the complex network method [23]. Song et al. examined the characteristics of the embodied carbon flow network among 30 sectors in China based on China’s input–output extension table in 2015 [17]. On the basis of a network perspective, Li et al. analyzed the global trade embodied carbon flow and community division in 2000 and 2015, which revealed the status and relationship evolution trends of different countries and regions in the global trade embodied carbon flow network [24]. The existing literature shows that the analysis of embodied carbon flow networks has primarily focused on the national scale, such as examining the characteristics of embodied material flow, embodied energy flow, and embodied carbon flow networks of trade between countries. For the application of the multiregional input–output analysis method to carbon emissions, it is necessary to comprehensively calculate the carbon emissions generated throughout the entire production chain, from the mining of raw materials to manufacturing to the final product. The research object is the carbon emissions caused by the specific production chain [25]. As the production chain is an orderly connection between upstream and downstream production links, the shape is a “chain”, and the products produced in each production process are accompanied by certain carbon emissions, that is, the products of each production link have embodied carbon. Multiregional input–output analysis involves tracing the embodied carbon through the entire production chain from upstream to downstream according to the production chain to the final product (products that no longer enter the production field). The complex network analysis method mainly investigates the network characteristics of embodied carbon flow in all production links from the perspective of the national economic industrial system. In the national economic system, all production chains have formed a larger industrial network under the connection of various production links, and the shape is a “network”. In this manner, the analysis of embodied carbon can not only involve the upstream and downstream parts of a particular production chain, but also span other production chains.

To sum up, comparative analysis shows that the multiregional input–output analysis method focuses on accounting for the embodied carbon of a single production chain, which can be within the same region or across multiple regions. In contrast, the complex network analysis method focuses on analyzing the embodied carbon characteristics of all production chains. The latter approach considers the overall industrial network, thus enabling a more comprehensive analysis to determine the network characteristics of complex systems and providing internal information regarding key system components, which is the primary advantage of this method. The global embodied carbon emissions on national level shows complex network characteristics [22–24], as the world’s largest embodied carbon emissions of export, what are the network characteristics of the carbon flow embodied in interprovincial trade of China? Therefore, this paper contributes in the following aspects: first, from the complex network analysis of carbon emissions embodied in global trade based on national scale to the complex network analysis of carbon emissions embodied in exports at the provincial level, the network analysis of at different spatial scales is further improved; second, based on the visual comparison of the carbon flows among China’s provincial industries in 1997, 2007 and 2017, this paper summarizes the relevant characteristics, and provides policy implications for the Chinese government to achieve the dual carbon goal implements at the provincial level.

The remaining contents are orgnized as follows. Section 2 introduces data sources and methods. Section 3 focuses on the analysis of networks of embodied carbon emissions caused by China’s export at the provincial level. Section 4 discusses and section 5 concludes.

## 2. Data sources and methods

### 2.1. Data sources

The CO_2_ emission data in this paper were obtained from the statistical yearbook of China’s energy consumption (2008–2018). The CO_2_ emissions were calculated by using factor coefficients such as the average low calorific value and carbon content per unit calorific value of various fossil energies in IPCC report. The input–output table data were from the multiregional input–output tables of China in 2007 and 2012 jointly compiled by the Institute of Geographical Sciences and Natural Resources Research and the National Bureau of Statistics of the Chinese Academy of Sciences [26, 27], and the multiregional input–output table of China in 2017 complied on the basis of Chinese regional input-output table of 31 provincial units in 2017 [28]. The study area was the 30 provinces and municipalities of mainland China not including Hong Kong, Macao, Taiwan, and Tibet. These industries of national economy were unified and merged into 27 sectors (Table 1).

**Table 1:** Numbers and description of Chinese industrial sectors.

Number	Industrial sector	Number	Industrial sector	Number	Industrial sector
1	Agricultural, forestry, animal husbandry, and fishery products and services	10	Paper printing and cultural, educational, and sporting goods	19	Communication equipment, computers, and other electronic equipment
2	Coal mining and beneficiation products	11	Petroleum, coking products, and nuclear fuel processing products	20	Instruments and apparatuses
3	Oil and gas extraction products	12	Chemical products	21	Other manufacturing
4	Metal ore mining and beneficiation products	13	Non-metallic mineral products	22	Production and supply of electric power and heat
5	Non-metallic ore and other ore mining and dressing products	14	Metal smelting and calendering products	23	Production and supply of gas and water
6	Food and tobacco	15	Metalware	24	Construction
7	Textiles	16	General and special equipment manufacturing	25	Transportation, storage, and postal service
8	Textile, clothing, shoes, hats, leather, down and their products	17	Transportation equipment	26	Wholesale, retail, accommodation, and catering
9	Wood products and furniture	18	Electrical machinery and equipment	27	Other services

### 2.2. Research methods

The research methods were mainly divided into two parts. The first part aimed to make a complete accounting of the embodied carbon for all industries in China’s provinces through multiregional input–output analysis. The second part aimed to construct the interprovincial embodied carbon flow network of China’s exports based on the mean threshold, then the network characteristics, key nodes and edges, and community structure of the interprovincial embodied carbon flow of China’s exports were studied by the complex network analysis method.

#### 2.2.1. Calculation of CO_2_ emissions embodied by exports between industrial sectors

According to input–output analysis theory, the classical Leontief formula can be expressed as follows:

AX+Y=X, (1)

where *A* is the direct consumption coefficient matrix, *X* is the total output column vector, and *Y* is the column vector of the region’s final demand (include consumption, export, and fixed capital formation). Further transforming the identity of Eq (1) gets Eq (2):

$$X={\left(I-A\right)}^{-1}Y.$$ (2)

The purpose of Eq (2) is to establish a linear relationship between the final demand and the total output through the Leontief inverse matrix. Because the Leontief inverse matrix reflects the current production technology level, it is generally considered to be relatively stable in the short term. Therefore, as long as the change in the final demand of each sector is determined, the total output driven by the final demand can be calculated. The CO_2_ emissions per unit of total output (*e*_*i*_) for sector *i* can then be determined from the total CO_2_ emissions (*C*_*i*_) and total output (*x*_*i*_), as shown in Eq (3), then a diagonal matrix can be introduced to obtain Eq (4):

$$e_{i}=\frac{C_{i}}{x_{i}},$$ (3)

$$E=\hat{e}∙{\left(I-A\right)}^{-1}\hat{Y}.$$ (4)

When the final demand is the export diagonal matrix $\hat{F}$, the embodied CO_2_ flow between sectors caused by the exports of each sector can be obtained:

$$E=\hat{e}∙{\left(I-A\right)}^{-1}\hat{F}.$$ (5)

#### 2.2.2. Analysis of the interprovincial embodied carbon flow network of china’s exports

*2*.*2*.*2*.*1*. *Construction of the interprovincial embodied carbon flow network of china’s exports*. Through Eq (5), the set of embodied CO_2_ flow relationships between exports of different sectors among provinces can be calculated and the interprovincial embodied carbon flow network of China’s exports can be constructed. The provincial industrial sectors are taken as the network nodes and the CO_2_ flow volumes are taken as the network edges. All nodes and edges constitute the interprovincial embodied CO_2_ flow network of China’s exports. The embodied CO_2_ flow network has two-way flow between the origin and destination. In this study, the inflow and outflow were added to obtain the undirected flow matrix R = [r_ij_], based on the average embodied carbon flow between different sectors $\overset{-}{r}$ as the critical value, and *R* was converted to bisection matrix *Z*; if r_ij_ was greater than or equal to $\overset{-}{r}$, the elements of matrix *R* were assigned a value of 1, otherwise they were assigned a value of 0. In this manner, the interprovincial embodied carbon flow network matrix of China’s exports was constructed through the mean threshold, which is expressed as Eq (6):

$$\overset{-}{r}=\frac{1}{n(n-1)}\sum_{i\neq j}r_{ij}.$$ (6)

*2*.*2*.*2*.*2*. *Network characteristic analysis*. Complex networks generally have scale-free and small-world characteristics. In a scale-free network, the degree distribution of nodes conforms to a power law, that is, the degree distribution is not uniform, the degree of a few nodes is very large, and the degree of most nodes is very small. The degree of a node generally refers to the number of connected edges, represented by *k*. The probability distribution function *f*(*k*) of the network node degree can be expressed as *f*(*k*) = *k*^−*θ*^. Taking logarithms of both sides affords Eq (7). Through the statistical analysis and testing of Eq (7), the scale-free characteristics of the network can be investigated.


$$lnf\left(k\right)=-\hat{\theta }lnk.$$ (7)

So-called small-world networks have the feature that although the network scale is very large, there exists a relatively short path between any two nodes, that is, generally speaking, the number of connections between a single node is very small, but it can connect the whole world. The characteristic path length and clustering coefficient are often used to reflect the small-world characteristics of complex networks [18]. The path length is the average of the shortest path length of all nodes in the network. The shortest path length (*d*_*ij*_) refers to the minimum number of edges to pass from node *i* to node *j*. If two nodes have no edges and are not connected, the shortest path length $d_{ij}$ is infinite. The path length of an undirected network (*L*) can be expressed as follows:

$$L=\frac{2}{n(n-1)}\sum_{i=1}^{n}\sum_{j=i+1}^{n}d_{ij}.$$ (8)

The clustering coefficient of a node (*C*_*i*_) refers to the ratio between the actual number of connected edges and the maximum possible number of connected edges between the node and the surrounding adjacent nodes. The clustering coefficient of a network is the average value of the clustering coefficients of all nodes, which represents the average interconnection probability between network nodes and can be expressed as follows [18]:

$$C=\frac{1}{n}\sum_{i=1}^{n}C_{i}.$$ (9)

*2*.*2*.*2*.*3*. *Key nodes and edges of a network*. The intermediary role of nodes is often measured by the intermediary number of nodes. The shortest paths between many non-adjacent nodes *j* and node *l* will often include a specific key node *i*. If this node *i* is traversed by many different shortest paths, then the node *i* is a key node in the network. The betweeness number *B*_*i*_ of the key node *i* can be calculated as shown in Eq (10) [18], where *N*_*jl*_(*i*) is the number of shortest paths for all non-adjacent nodes *j* and node *l* passing through node *i* and *N*_*jl*_ is the number of shortest paths for all non-adjacent nodes *j* and node *l*:

$$B_{i}=\sum_{i\neq j\neq l}N_{jl}(i)/N_{jl}.$$ (10)

Similarly, the mediating effect of edges can also be measured by the number of edges. When many non-adjacent nodes in the network through the shortest path, they will pass through a specific edge *e*_*im*_ with the betweeness number *B*_*im*_ as defined in Eq (11) [18]. Here, *N*_*jl*_(*e*_*im*_) is the number of shortest paths for all non-adjacent nodes *j* and node *l* passing through edge *e*_*im*_ and *N*_*jl*_ is the number of shortest paths for all non-adjacent nodes *j* and node *l*.


$$B_{im}=\sum_{j\neq l\;and\left\{jl\right\}\neq \{im\}}N_{jl}(e_{im})/N_{jl}.$$ (11)

*2*.*2*.*2*.*4*. *Network community partition algorithm*. Some nodes in a network are closely related and form a relatively independent community. Dividing the community in the network helps to analyze the internal key structure of the network, but the community division algorithm is not unique. According to the constructed interprovincial embodied carbon flow network of China’s exports, the Louvain algorithm was adopted. This algorithm is based on the multilayer optimization of modularity. Each node and nearby nodes join the community one by one to find the maximum modularity gain and achieve the optimal division. This approach has the advantage of fast calculation speed when dealing with large networks [29]. The modularity (*Q*) is a widely used index to evaluate the quality of community division; this index compares the internal node connection edges of the divided network community with random connections, where a larger value of *Q* indicates a superior division result:

$$Q=\frac{1}{2M}\sum_{i,j}\left(\tau_{ij}-\frac{k_{i}k_{j}}{2M}\right)\delta \left(\sigma_{i},\sigma_{j}\right).$$ (12)

Here, *M* is the number of edges in the network and *τ*_*ij*_ is a network adjacency element. If there is a connection between node *i* and node *j*, then *τ*_*ij*_ = 1; otherwise *τ*_*ij*_ = 0. *k*_*i*_ and *k*_*j*_ are the degrees of node *i* and node *j*, respectively. *δ*(*σi*, *σ*_*j*_) is a membership function. When node *i* and node *j* belong to the same community, that is, when *σi* = *σ*_*j*_, then *δ*(*σi*, *σ*_*j*_) = 1 otherwise *δ*(*σi*, *σ*_*j*_) = 0.

As the basic unit of the network, the community can also be analyzed by several indicators [30]. For example, Eq (13) defines the community aggregation coefficient (*ρ*), which measures the number of nodes in the community; the greater the community aggregation coefficient, the more nodes in the community. Here, $V_{P_{s}}$ is the node set that divides the network into community *P*_*s*_, and *V* is all the node sets in the network. It is easy to determine from Eq (13) that the sum of the aggregation coefficients of all communities is equal to 1.


$$\rho =\frac{V_{P_{s}}}{V}.$$ (13)

The correlation density coefficient (*μ*) for the community *P*_*s*_ is defined in Eq (14), which measures the association status within the community *P*_*s*_. The greater the correlation density coefficient of the community *P*_*s*_, the greater correlation density within the community *P*_*s*_. Similarly, it can be obtained from Eq (14) that the sum of the correlation density coefficients of all communities is equal to 1.


$$\mu =\frac{\sum_{j,l\in P_{s}}\omega_{jl}}{\sum_{j,l}\omega_{jl}}.$$ (14)

Further investigate the degree of connection between communities and node *j* and node *l* belong to different communities *P*_*s*_ and *P*_*t*_, where *ω*_*jl*_ is the edge weight of node *j* and node *l*. If there are *θ* communities, then the connection coefficient *τ* between the whole network community is as follows:

$$\tau =\frac{2}{\theta \left(\theta -1\right)}\sum_{j\epsilon P_{s},l\epsilon P_{t}}\omega_{jl}.$$ (15)

## 3. Results

### 3.1. Characteristics of China’s interprovincial embodied carbon flow network

**Fig 1:**
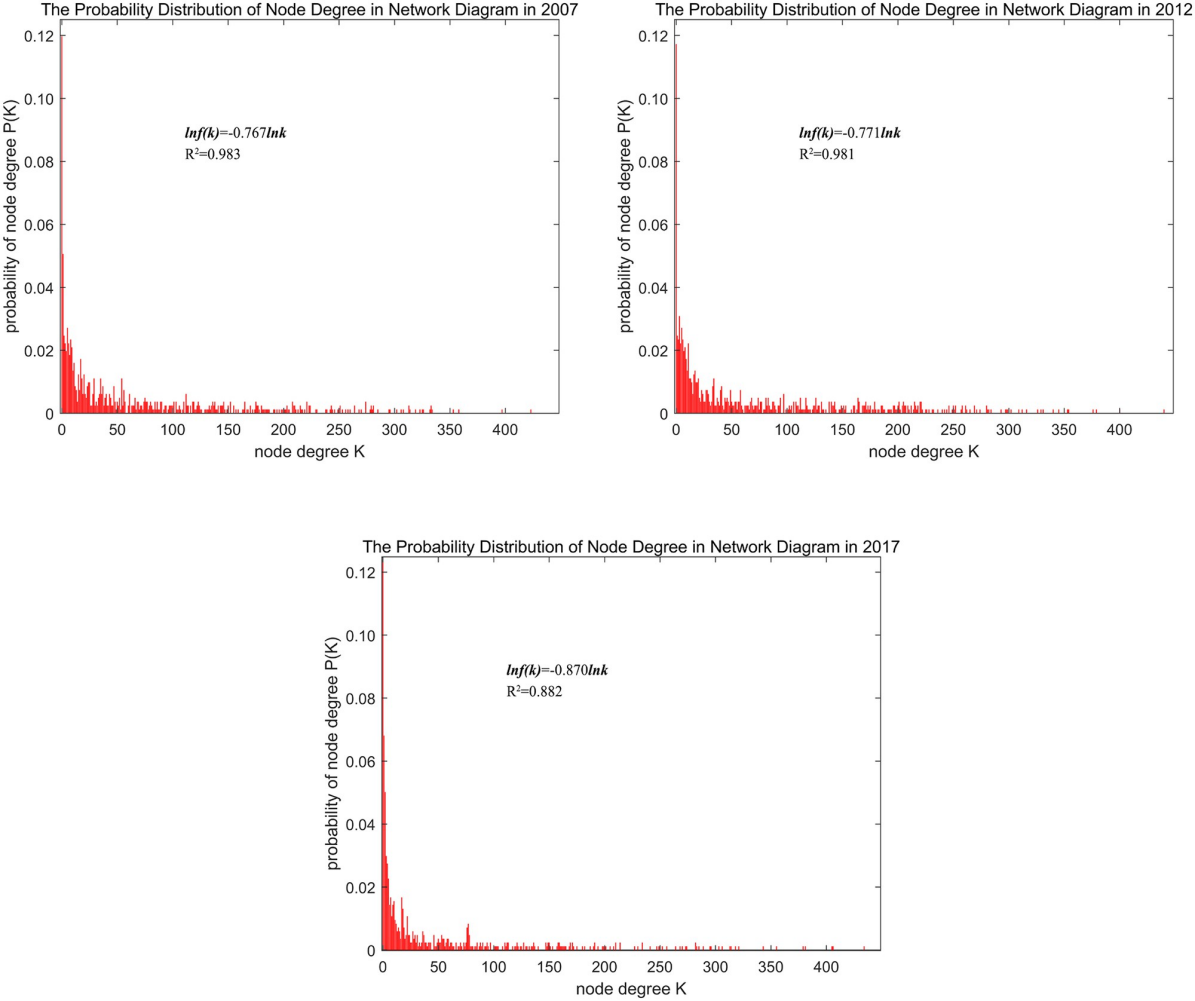
Node degree probability distributions in the network diagrams for 2007, 2012 and 2017.

Through statistical analysis based on Eq (7), the node degree probability distribution curves for the interprovincial embodied carbon flow networks of China’s exports in 2007, 2012 and 2017 were obtained (Fig 1). The goodness-of-fit values were 0.98, 0.98 and 0.88 for three curves, which obviously conforms to a decreasing power law distribution, that is, the nodes with large degree values (number of connecting edges) accounted for a minor proportion and the nodes with small degree values accounted for a major proportion. In 2007, 2012 and 2017, the power exponents in logarithmic form were −0.767, −0.771 and -0.811, respectively, while the t values of the regression coefficients were −112.1, −110.1 and -115.4, respectively, the coefficients were significant at 1% significant level, indicating that the networks had scale-free characteristics. For the interprovincial embodied carbon flow network of China’s exports, the greater the degree of nodes in the provincial industrial sector, the greater the embodied carbon flow between the nodes. The scale-free characteristics of the carbon emission network mean that a large amount of embodied carbon flows through only a few nodes, which also shows that a few industrial sectors in the interprovincial embodied carbon flow network carry a large amount of embodied carbon; these nodes are the key nodes to reduce the embodied carbon of the entire network.

The characteristic path length in a complex network reflects the average distance between nodes in the whole network. The shorter the characteristic path length, the shorter the distance between nodes. According to calculations based on Eqs (8) and (9), the characteristic path lengths of the embodied carbon flow networks between provincial industrial sectors in 2007, 2012 and 2017 were 1.69, 1.65 and 1.87, respectively, indicating that the network nodes can be reached in about two steps and fewer edges. The clustering coefficients were 0.5482, 0.5421 and 0.5501, respectively, reflecting strong node connectivity characteristics. From the perspective of the short characteristic path length and large clustering coefficient, the interprovincial embodied carbon flow networks of China’s exports in 2007, 2012 and 2017 exhibited significant small-world characteristics and corresponded to small-world networks. Because the interprovincial embodied carbon flow networks of China’s exports displayed small-world characteristics, the average number of edges observed for the network node connections means that the industrial sectors of each province have fewer production links. Thus, when a provincial industrial sector implements carbon emission reduction measures, it can quickly affect other industrial sectors; at the same time, the strong node connectivity characteristics show that the degree of correlation between provincial industrial sectors is very high. Carbon emission reduction by a provincial industrial sector can affect many related industrial sectors. By affecting key nodes in the network, the effect of “four or two pulling a thousand pounds” can be realized.

### 3.2. Key nodes and edges of China’s interprovincial embodied carbon flow network

It can be seen from the previous analysis that the scale-free characteristics of the embodied carbon flow network indicate that there are a large number of embodied carbon flows between a few nodes. Therefore, by identifying the key nodes and edges in the complex network, we can analyze the key provincial industrial sectors involved in the embodied carbon flow and the production chain formed between sectors. These key provincial industrial sectors and the production chain formed between sectors are a small number of nodes and edges with a large amount of embodied carbon in the network. Betweeness is a commonly used concept in complex network analysis. They are used to express the role of “bridges” in a network and play an important part in the process of network construction. When a “bridge” node or edge is lost, it can paralyze the entire network. Therefore, the greater the intermediary role of this node, the more critical the node or edge in the whole network. The key nodes and edges of the embodied carbon flow networks in 2007, 2012 and 2017 were calculated according to Eqs (10) and (11) (Tables 2 and 3).

**Table 2:** Top ten key node sectors of the embodied carbon flow networks of China’s exports.

Rank	Key nodes in 2007	Key nodes in 2012	Key nodes in 2017
1	Metal smelting and calendering products in Hebei	Chemical products in Hebei	Metal smelting and calendering products in Hebei
2	Petroleum, coking products, and nuclear fuel processing products in Shanxi	Communication equipment, computers, and other electronic equipment in Jiangsu	Other services in Beijing
3	Electrical machinery and equipment in Guangdong	Petroleum, coking products, and nuclear fuel processing products in Hebei	Chemical products in Hebei
4	Communication equipment, computers, and other electronic equipment in Guangdong	Metal smelting and calendering products in Qinghai	Metal smelting and calendering products in Shandong
5	Textile, clothing, shoes, hats, leather, down and their products in Guangdong	Chemical products in Shandong	Chemical products in Shandong
6	Textiles in Jiangsu	Chemical products in Jiangsu	Chemical products in Hubei
7	Metal smelting and calendering products in Guangdong	Production and supply of electric power and heat in Inner Mongolia	Metal smelting and calendering products in Shanghai
8	General and special equipment manufacturing in Guangdong	Transportation, storage, and postal service in Shanghai	Chemical products in Inner Mongolia
9	Production and supply of electric power and heat in Inner Mongolia	Production and supply of electric power and heat in Shanxi	Metal smelting and calendering products in Liaoning
10	Production and supply of electric power and heat in Henan	Communication equipment, computers, and other electronic equipment in Guangdong	Transportation, storage, and postal service in Beijing

**Table 3:** Top ten key edges of the embodied carbon flow networks of China’s exports.

Rank	Key edges in 2007	Key edges in 2012	Key edges in 2017
1	Oil and gas extraction products in Hubei–Chemical products in Hubei (intraprovince)	Chemical products in Hebei–Metal smelting and calendering products in Jilin (interprovince)	Other services in Beijing-Chemical products in Hebei (interprovince)
2	Production and supply of electric power and heat in Ningxia–Other manufacturing in Ningxia (intraprovince)	Production and supply of electric power and heat in Jilin–other manufacturing in Jilin (intraprovince)	Transportation, storage, and postal service in Beijing-Metal smelting and calendering products in Hebei (interprovince)
3	Production and supply of gas and water in Henan–Textile, clothing, shoes, hats, leather, down, and their products in Guangdong (interprovince)	Oil and gas extraction products in Gansu–Transportation, storage, and postal services in Shanghai (interprovince)	Communication equipment, computers, and other electronic equipment in Chongqing–Other services in Beijing (interprovince)
4	Production and supply of electric power and heat in Jilin–Other manufacturing in Jilin (intraprovince)	Production and supply of electric power and heat in Jilin–Chemical products in Hubei (interprovince)	Other services in Beijing–Production and supply of gas and water in Hebei (interprovince)
5	Petroleum, coking products, and nuclear fuel processing products in Heilongjiang–Transportation, storage, and postal service in Heilongjiang (intraprovince)	Petroleum, coking products, and nuclear fuel processing industry in Heilongjiang–Transportation, storage, and postal services in Heilongjiang (intraprovince)	Chemical products in Inner Mongolia–Production and supply of electric power and heat in Inner Mongolia (intraprovince)
6	Textiles in Jiangsu–Textiles in Zhejiang (interprovince)	Non-metallic ore and other ore mining and dressing products in Guangdong–Chemical products in Jiangsu (interprovince)	Transportation, storage, and postal service in Tianjin–Construction in Hebei (interprovince)
7	Production and supply of gas and water in Fujian–Textiles in Fujian (intraprovince)	Chemical products in Shandong–Construction in Shandong (intraprovince)	Chemical products in Shandong–Metal ore mining and beneficiation products in Beijing (interprovince)
8	Production and supply of gas and water in Fujian–Chemical industry in Hubei (interprovince)	Production and supply of electric power and heat in Shaanxi–Metal ore mining and beneficiation products in Shaanxi (intraprovince)	Chemical products in Shandong–Chemical products in Inner Mongolia (interprovince)
9	Production and supply of gas and water in Zhejiang–Textiles in Guangdong (interprovince)	Metal ore mining and beneficiation products in Jiangxi–Metal smelting and calendering products in Jiangxi (intraprovince)	General and special equipment manufacturing in Hunan–Metal smelting and calendering products in Hunan (intraprovince)
10	Metal ore mining and beneficiation products in Hainan–Metal smelting and calendering products in Guangdong (interprovince)	Production and supply of gas and water in Hubei–Transportation, storage, and postal service in Hubei (intraprovince)	Non-metallic mineral products in Fujian–Non-metallic mineral ore and other ore mining and dressing in Shandong (interprovince)

In the embodied carbon flow network composed of 27 sectors in 30 provinces underlying China’s exports, among the top ten key node sectors in 2007, there are three energy production and supply sectors (e.g., petroleum, nuclear fuel, power and heat production), the other seven sectors were manufacturing (including the metal smelting and calendering products in Hebei, the electrical machinery and equipment in Guangdong, the communication equipment, computers, and other electronic equipment in Guangdong, the textile, clothing, shoes, hats, leather, down, and their products in Guangdong, the textiles in Jiangsu, the metal smelting and calendering products in Guangdong, and the general and special equipment manufacturing in Guangdong). In addition to the three energy production and supply sectors including the petroleum, coking products, and nuclear fuel processing in Hebei, the production and supply of electric power and heat in Inner Mongolia, and the production and supply of electric power and heat in Shanxi, the other seven key node sectors in 2012 comprised six manufacturing sectors (including the chemical products in Hebei, the communication equipment, computers, and other electronic equipment in Jiangsu, the metal smelting and calendering products in Qinghai, the chemical products in Shandong, the chemical products in Jiangsu, and the communication equipment, computers, and other electronic equipment in Guangdong) and a service sector, namely, the transportation, storage, and postal services in Shanghai. The ten key node sectors in 2017 are mainly manufacturing sectors, which include the metal smelting and calendering products in Hebei, Shandong, Shanghai and Liaoning, chemical products in Hebei, Hubei, Shandong and Inner Mongolia. Besides, there are also transportation, storage, and postal service and other services in Beijing. With respect to the time evolution of the key network node sectors, first, the categories of industrial sectors in 2007 were relatively simple, with only two categories: the energy production and supply sector and the manufacturing sector. In 2012, the service sector was newly added. In 2012, the proportion of China’s GDP originating from the service industry exceeded that originating from secondary industry for the first time and the former became the main driver of economic growth. The embodied carbon due to the service industry also increased, and the transportation, storage, and postal service has now emerged as a sector that should be concerned about carbon emission reduction. For key node sectors in 2017, the proportion of services as key node has been increased. Second, in 2007, the provinces where the energy production and supply sectors were located were distributed in the central and western regions, and the provinces where the manufacturing sectors were located were all distributed in the Southern regions, primarily Guangdong. This spatial distribution reflects the regional division of labor between the central and western regions responsible for providing energy resources and the eastern regions responsible for production and manufacturing. The embodied carbon flow also shows a pattern of flowing from the central and western regions to the eastern region. By 2012, in addition to the central and western regions, the energy production and supply sector had also appeared in the eastern region, and the provinces where the manufacturing sector was located were not only mainly concentrated in the eastern region but also newly added. The spatial layout of the key nodes of the embodied carbon flow in the western region had also changed, and petroleum processing in Hebei in the east and metal smelting in Qinghai in the west had become key node industries. By 2017, the key nodes in eastern provinces have increased, which mainly distributed in Beijing, Hebei and Shandong. Overall, compared with the energy production and supply industry in the central and western regions and manufacturing industry in the eastern region in 2007, in terms of the obvious spatial distribution pattern of key nodes, the distribution of network key nodes in industrial sectors and provinces in 2012 and 2017 showed more diversified characteristics, which indicates that the low-carbon measures required for China’s exports were also facing a more complex situation.

From the top ten key edges of the network, there were five production chains within provinces and five production chains between provinces in 2007, of which seven were between energy production and supply sectors and manufacturing sectors (the oil and gas extraction products in Hubei and the chemical products in Hubei, Production and supply of electric power and heat in Ningxia and other manufacturing in Ningxia, production and supply of gas and water in Henan and the textile, clothing, shoes, hats, leather, down and their products in Guangdong, production and supply of electric power and heat in Jilin and other manufacturing in Jilin, the production and supply of gas and water in Fujian and the textiles in Fujian, the production and supply of gas and water in Fujian and the chemical products in Hubei, and the production and supply of gas and water in Zhejiang and the textiles in Guangdong). In addition, there was a chain between manufacturing sectors (the textiles in Jiangsu and the textiles in Zhejiang), a chain between a resource mining sector and a manufacturing sector (the metal ore mining and beneficiation products in Hainan and the metal smelting and calendering products in Guangdong), and a chain between an energy production and supply sector and a service sector (the petroleum, coking products, and nuclear fuel processing products in Heilongjiang and the transportation, storage, and postal service in Heilongjiang). In 2012, there were six intraprovince production chains and four interprovince production chains, of which the chains between energy production and supply sectors and manufacturing sectors had reduced to only two (the production and supply of electric power and heat in Jilin and other manufacturing in Jilin, and the production and supply of electric power and heat in Jilin and the chemical products in Hubei), while the chains between energy production and supply sectors and service sectors had increased to three (the oil and gas extraction products in Gansu and the transportation, storage, and postal service in Shanghai, the petroleum, coking products, and nuclear fuel processing products in Heilongjiang and the transportation, storage, and postal service in Heilongjiang, and the production and supply of gas and water in Hubei and the transportation, storage, and postal service in Hubei) and the chains between resource exploitation sectors and manufacturing sectors had also increased to two (the non-metallic ore and other ore mining and dressing products in Guangdong and the chemical products in Jiangsu, and the metal ore mining and beneficiation products in Jiangxi and the metal smelting and calendering products in Jiangxi). The remainder include a chain between manufacturing sectors (the chemical products in Hubei and the metal smelting and calendering products in Jilin), a chain between a manufacturing sector and a construction sector (the chemical products in Shandong and the construction in Shandong), and a chain between an energy production and supply sector and a resource mining sector (the production and supply of electric power and heat in Shaanxi and the metal ore mining and beneficiation products in Shaanxi). In 2017, intra-provincial production chains are reduced to two chains, inter-provincial production chains are increased to eight chains.

According to these changes over time, the intraprovincial production chains were primarily distributed in the central and western regions, mainly from energy production and supply sectors to manufacturing sectors, while the interprovincial production chains were mainly distributed in the eastern region, mainly from manufacturing sectors to manufacturing sectors, indicating that the production chains with high embodied carbon in the central and western regions were mainly related to energy production and supply. In 2007, most of the chains were between energy production and supply sectors and manufacturing sectors, whereas in 2012, the chains between energy production and supply sectors and service sectors were predominant. The chains between other types of resource mining sectors and manufacturing sectors were also prominent, and manufacturing sectors and construction sectors were newly added. According to data from the China Statistical Yearbook [31], the construction industry accounted for 6.9% of national GDP in 2012, corresponding to a peak period. The construction industry in Shandong province accounted for 8.2% of the national total, second only to Jiangsu. The construction industry has a large demand for highly energy-consuming products such as steel bars, cement, and glass, resulting in the construction industry in Shandong province becoming a part of the key carbon production chain in the network. In 2017, key production chains of interprovincial have increased. These changes also once again confirm the key production chain behind China’s exports from 2007 to 2017 has become more diversified.

### 3.3. Community characteristics of China’s interprovincial embodied carbon flow network

**Fig 2:**
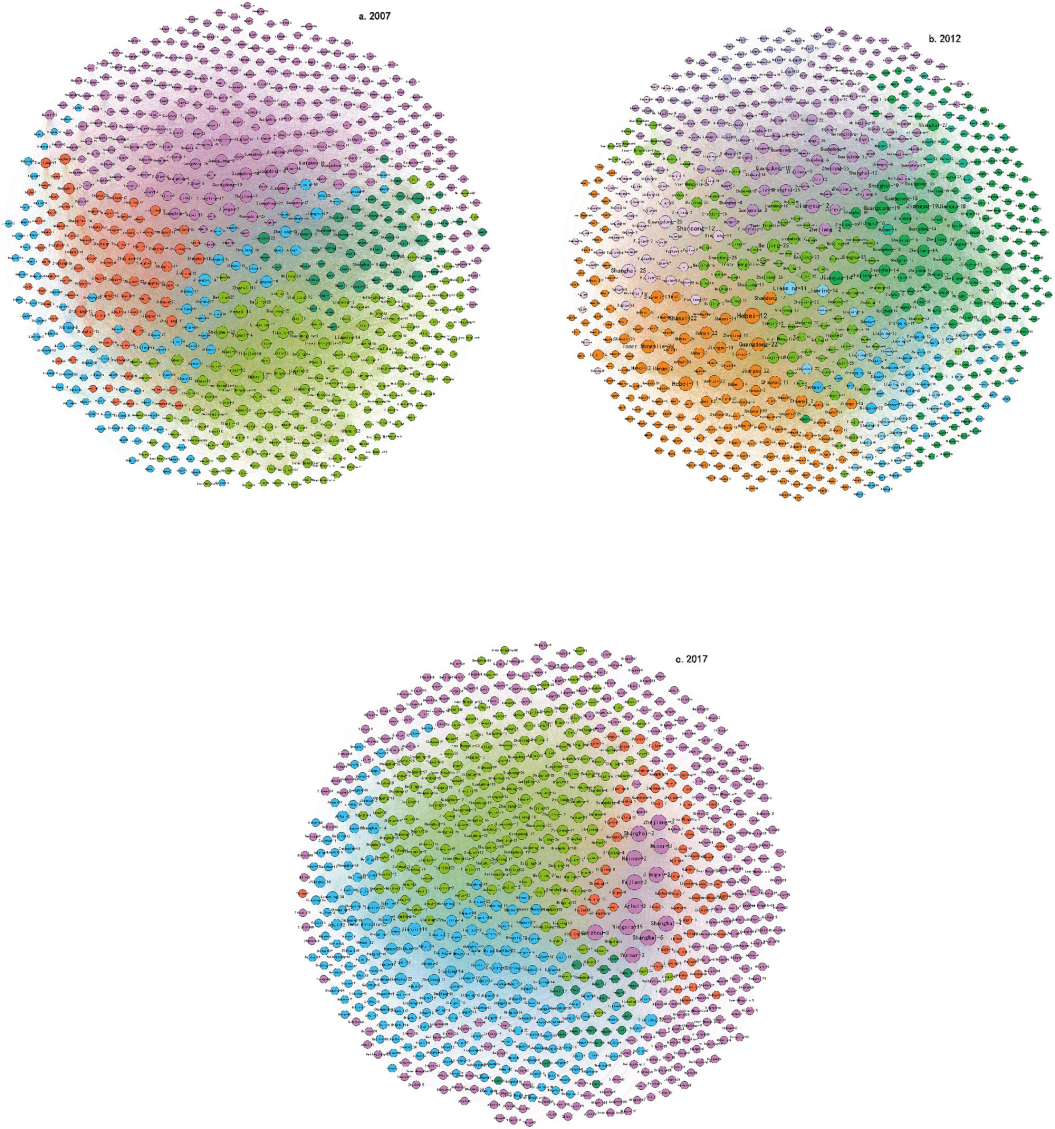
Community division of embodied CO_2_ flow network results of China’s exports in 2007, 2012 and 2017.

**Fig 3:**
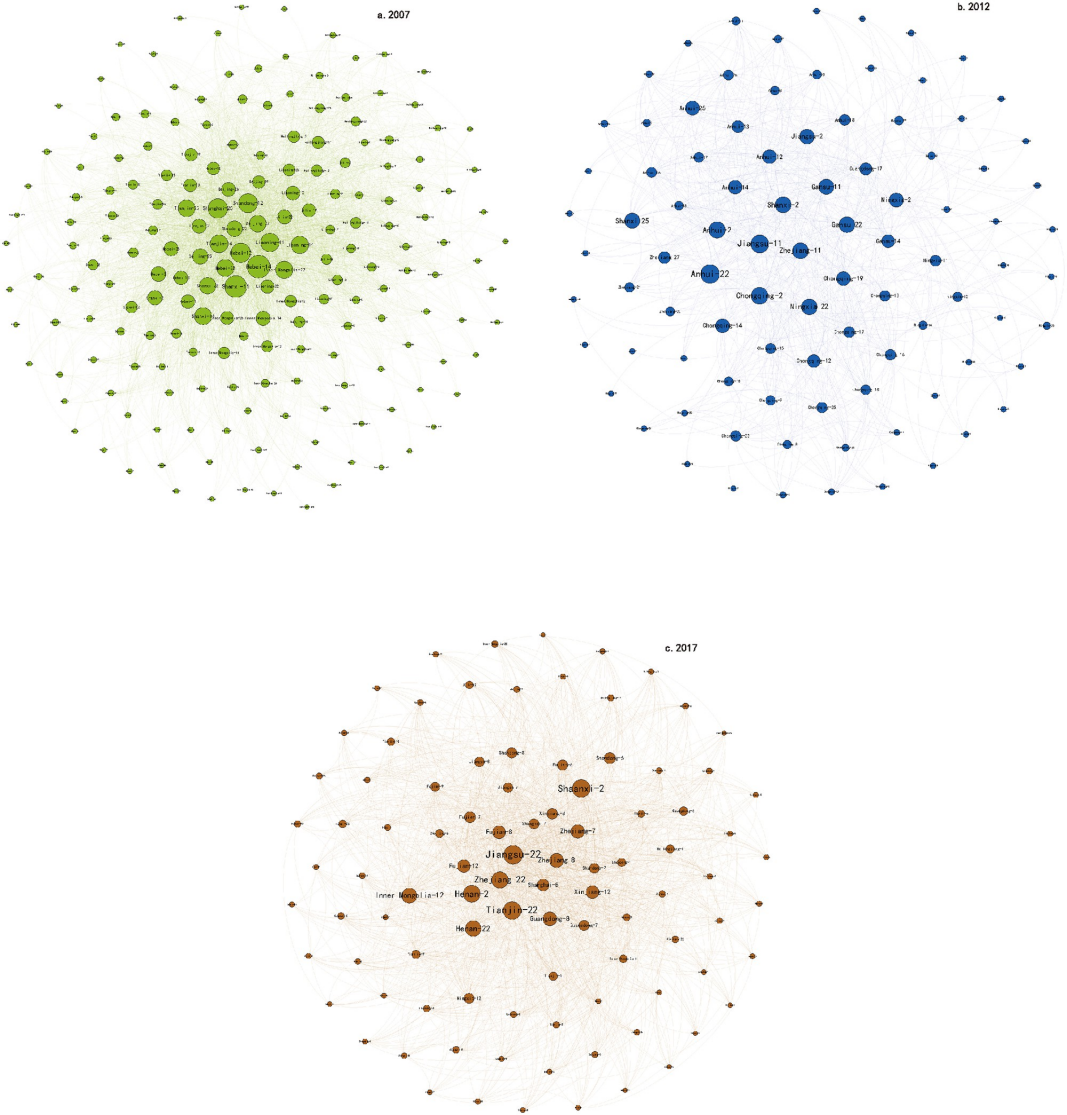
First network communities in 2007, 2012 and 2017.

According to Eq (12), the maximum modularity in 2007 was 0.139, which was divided into five communities (Fig 2), the average value of aggregation coefficient of which is 0.66. Compare to 2007 it was 0.128 in 2012, the phenomenon of small network agglomeration and dispersion was more prominent, which was divided into 11 communities (Fig 2), the average aggregation coefficient of all communities was 0.63. In 2017, the maximum modularity was 0.194, and the average aggregation coefficient of all communities was 0.77, which was divided into five communities (Fig 3). The average value of the network community aggregation coefficient in 2007 was higher than that in 2012, indicating that from 2007 to 2012, the aggregation degree of nodes in the network decreased, that is, the number of sector nodes associated with a single community decreased, while from 2012 to 2017, the aggregation degree of nodes between networks increased slowly, showing that the links between communities were strengthened, and the carbon emissions of trade is concentrated to a few sector nodes. From the perspective of spatial distribution, the embodied carbon flow network of China’s inter-provincial trade has always been in a state of close internal links. Under the influence of the Belt and Road Initiative and RCEP, foreign trade exports have ushered in a broader development. At the same time, under the Rise of the Central China, the coordinated development of Beijing, Tianjin and Hebei etc., the diversification of communities within the embodied carbon emissions of China’s interprovincial trade.

Further investigate the contents of the communities divided. Owing to the large number of communities involved and limited space, we randomly investigated one of the communities in 2007, 2012 and 2017. Taking the first community divided in 2007 as an example, it is mainly reflected in the industrial association of northern provinces and other provinces (Fig 3a). In addition to these industrial clusters formed by industrial association within the province, the industrial clusters formed by industrial association between the provinces were also more obvious, such as the production and supply of electric power and heat industry in Shandong and the Other services industry in Beijing and the metal ore mining and beneficiation products industry in Tianjin and the chemical products industry in Hebei. The community aggregation coefficient and the association density of this community is 0.17 and 0.15. Similarly, the community aggregation coefficient of the first community in 2012 the association density of the community is 0.15. It is mainly reflected in the industrial association of central provinces and other provinces (Fig 3b), such as the textile, clothing, shoes, hats, leather, down and their products industry in Ningxia and the petroleum, coking products, and nuclear fuel processing products industry in Zhejiang. The first community in 2017 is the industrial relation of Pearl River Delta and Yangtze River Delta. Such as the textiles industry in Zhejiang and the Production and supply of electric power and heat industry in Jiangsu and the textile, clothing, shoes, hats, leather, down and their products industry in Fujian and the textiles industry in Shanghai. The community aggregation coefficient and the association density of this community is 0.13 and 0.04, industrial chain of textile, clothing, shoes, hats, leather, down and their products plays a pivot role in this community. Compare to 2007 and 2012, the first community of 2017 is more centralized, the agglomeration effect of key provinces and sectors is further highlighted according to the changes of the average value of aggregation coefficient.

From the division of the interprovincial embodied carbon flow network underlying China’s exports, it can be seen that the number of communities in the embodied carbon flow network increased from 2007 to 2012 and then decreased from 2012 to 2017, while the correlation density coefficient and inter-association coefficient showed corresponding changes. Compared with the feature of more concentration and polarization of the interprovincial embodied carbon in the network for 2007, the feature was more decentralized and equal of the interprovincial embodied carbon flow in 2012, which also confirms that the production chain shown in the previous key side conclusion is more diversified, however, concentration and polarization of the interprovincial embodied carbon in the network for 2017 again.

## 4. Discussion

As for China’s provincial inter-industry embodied carbon flows, most of studies focused on input-output analysis [32, 33], this paper constructs an inter-provincial inter-industry carbon flow network based on interprovincial input-output database, and analyzes the key provinces and key sectors that affect embodied carbon emissions of export from the perspective of network analysis. The results show that the provincial and sectoral distribution of embodied carbon emissions of export are uneven, a few provinces and sectors carry a large amount of embodied carbon, which is concentrated in major coastal provinces (such as Guangdong and Jiangsu) and major energy consumption provinces (such as Inner Mongolia and Shanxi), which is consistent with the research conclusions of recent literatures [32, 33]. The contribution of this paper is to further refine the analysis of the complex network of embodied carbon to provincial level. By identifying the key "bridges" in the network, determine the main flow path of embodied carbon. Based on the network community detection, it is found that the node and correlation density within and between communities are declining from 2007 to 2012, which implies that carbon flow of inter-provincial trade is more decentralized and equal. Using the method of network analysis makes the spatial distribution of trade implied carbon flow more intuitive, and also makes the bridge that affects the key path of national export implied carbon clearer. This is the advantage of network analysis. Compared with input-output analysis applied to the accounting of provincial direct or indirect carbon emissions, the network analysis method is more clear for finding the key provinces and industries that need to be determined for national carbon emission reduction.

However, there are also some uncertainties in the network analysis of embodied carbon calculated based on the input-output method. First, with regard to the network construction, thresholds such as the average value are set according to the edges of the embodied carbon flow, and only edges greater than or equal to the threshold are retained in the network. However, different standard settings of threshold may also cause different results. Second, the selection method of key nodes and edges in the network is not absolute. Similarly, there may be differences in determining some key nodes and edges. However, for the investigation of embodied carbon emissions of trade, whether there may be differences in the construction of networks or the differences in the setting methods of key nodes and edges, the embodied carbon with larger trade flow between the two regions and the nodes passing through more shortest paths are always more likely to become key edges and nodes. It can be seen that using network analysis to analyze the embodied carbon emissions of exports at the provincial level is a further in-depth analysis of embodied carbon based on the input-output method.

## 5. Conclusions and policy implications

### 5.1. Conclusions

The network analysis of international trade embodied carbon flow in previous literature mainly focused on the national scale. Owing to the existence of regional economic ties, the interprovincial embodied carbon flow behind China’s exports is also the same. The data set forms a structure similar to a complex network. On the basis of China’s interprovincial and interregional input–output tables for 2007, 2012 and 2017, the embodied carbon flow of various industrial sectors caused by China’s exports was calculated. As such, the interprovincial embodied carbon flow network of China’s exports was constructed by the dichotomy matrix method, and the overall structure of the embodied carbon flow network was analyzed with the aid of complex network technical indicators. The main conclusions are as follows: (1) Analysis of the overall network characteristics revealed that the interprovincial embodied carbon flow network of China’s exports has small-world and scale-free characteristics, which is in accordance with the basic characteristics of complex networks. The node degree probability distribution curves for the networks obviously conformed to a decreasing power law distribution, which shows that a few industrial sectors carry a large amount of embodied carbon and suggests that reducing the embodied carbon associated with China’s exports can achieve twice the results with half the effort as long as we pay attention to a few key sectors. (2) Determination of the key nodes and key edges in the networks demonstrated that industrial sectors and production chains such as the power and heat production and supply industry, the petroleum processing, coking, and nuclear fuel processing industry, and the metal smelting and rolling processing industry play the role of key “bridges” in the entire network, among which Guangdong, Hebei, Jiangsu, Inner Mongolia, and Shanxi are important node provinces and the main flow paths for the generation of embodied carbon in national exports. (3) Analysis of the network divided communities from 2007 to 2017 indicated that the general trend of the number of communities firstly increased then declining while of the aggregation coefficient of the node and correlation density within first community also firstly downward then upward, reflecting firstly decentralization then centralization of the interprovincial embodied carbon flow.

### 5.2. Policy implications

Analysis of the interprovincial embodied carbon flow network behind China’s exports confirmed that a few industrial sectors carry a large amount of embodied carbon, which means that implementing carbon emission reduction strategies could yield twice the results with half the effort as long as we grasp the key industrial sectors and implement node-to-area management by determining the network “bridges”. The identification of a key node or key edge means that the relevant industrial sectors or production chains participate in the total embodied carbon generated by most industrial sectors in the network, especially Guangdong, Hebei, Jiangsu, Inner Mongolia, and Shanxi etc. Therefore, these provincial industrial sectors or production chains should bolster their policies to encourage energy conservation and emission reduction, implement technological innovations, and ultimately reduce their carbon emissions, so as to reduce the embodied carbon of national exports on a large scale. From the key nodes and edges in the network in 2007, the types of key industries were single, and they became more diversified in 2012, especially in the service sector (transportation, warehousing, and postal industry) in many provinces. In 2012, the proportion of China’s GDP attributable to the tertiary industry reached 45.5%, thus exceeding that originating from the secondary industry (45.4%) for the first time. In 2017, the proportion of service sector has increased to 52.7% [31]. With China’s entry into the late stage of industrialization, the prominence of the service industry will continue to rise in the future. This is an inevitable development trend in the evolution of the industrial structure, which also reflects the deepening of professional division of labor and the extension of production chains. Thus, paying attention to carbon emission reduction in the service industry is the key to future low-carbon exports. In addition, the key nodes in the network for 2007 corresponded to the energy production and supply industry in the central and western regions and the manufacturing industry in the eastern region. In 2012, there were also manufacturing industries in the western regions and energy production and supply industries in the eastern regions, moreover, in 2017, the role of the service industry has gradually become prominent. With the implementation of the Belt and Road Initiative, the central and eastern regions will become more open to the outside world, and the key industrial sectors for carbon emission reduction will also show the trend of “deregionalization” and “focus on services” in the future.
